# Inhibition of complement activation by CD55 overexpression in human induced pluripotent stem cell derived kidney organoids

**DOI:** 10.3389/fimmu.2022.1058763

**Published:** 2023-01-12

**Authors:** Lonneke H. Gaykema, Rianne Y. van Nieuwland, Mette C. Dekkers, Mieke F. van Essen, Sebastiaan Heidt, Arnaud Zaldumbide, Cathelijne W. van den Berg, Ton J. Rabelink, Cees van Kooten

**Affiliations:** ^1^ Department of Internal Medicine-Nephrology, Leiden University Medical Center, Leiden, Netherlands; ^2^ Department of Cell and Chemical Biology, Leiden University Medical Center, Leiden, Netherlands; ^3^ Department of Immunology, Leiden University Medical Center, Leiden, Netherlands; ^4^ Eurotransplant Reference Laboratory, Leiden University Medical Center, Leiden, Netherlands; ^5^ The Novo Nordisk Foundation Center for Stem Cell Medicine (reNEW), Leiden University Medical Center, Leiden, Netherlands

**Keywords:** complement, CD55 (DAF), iPSCs, kidney organoids, CRISPR-Cas9, transplantation, immune modulation

## Abstract

End stage renal disease is an increasing problem worldwide driven by aging of the population and increased prevalence of metabolic disorders and cardiovascular disease. Currently, kidney transplantation is the only curative option, but donor organ shortages greatly limit its application. Regenerative medicine has the potential to solve the shortage by using stem cells to grow the desired tissues, like kidney tissue. Immune rejection poses a great threat towards the implementation of stem cell derived tissues and various strategies have been explored to limit the immune response towards these tissues. However, these studies are limited by targeting mainly T cell mediated immune rejection while the rejection process also involves innate and humoral immunity. In this study we investigate whether inhibition of the complement system in human induced pluripotent stem cells (iPSC) could provide protection from such immune injury. To this end we created knock-in iPSC lines of the membrane bound complement inhibitor CD55 to create a transplant-specific protection towards complement activation. CD55 inhibits the central driver of the complement cascade, C3 convertase, and we show that overexpression is able to decrease complement activation on both iPSCs as well as differentiated kidney organoids upon stimulation with anti-HLA antibodies to mimic the mechanism of humoral rejection.

## Introduction

End stage renal disease is increasing in prevalence worldwide. The inadequate availability of donor organs is the critical limiting factor in curative treatment. Human induced pluripotent stem cells (iPSCs) are a promising approach for regenerative medicine to circumvent the shortage of organ donors. Lineage specific differentiation protocols allow for the generation of many tissues, including kidney. Our group and others have shown that iPSC-derived kidney organoids contain nephrons and are vascularized upon transplantation (1–4), indicating the potential use of these organoids in transplantation to improve kidney function in patients. The use of autologous iPSC-derived tissues is not feasible in most cases, but transplantation of allogeneic tissue has an increased risk of rejection because of human leukocyte antigen (HLA) mismatches. Currently several strategies have been envisaged to generate hypoimmunogenic stem cells. While a lot of effort has been made to prevent T cell mediated rejection by disrupting HLA expression or by the overexpression of inhibitory molecules (5–8), no studies have been conducted to limit the deleterious effect of complement activation, while it has a significant role in graft rejection.

The complement system is part of the innate immune system and provides protection against micro-organisms. Activation of the cascade can be initiated *via* three separate routes: *via* binding of C1q to the Fc domains of surface bound antibodies (classical pathway), *via* mannose-containing polysaccharides recognition (lectin pathway) or *via* spontaneous hydrolysis of the internal C3 thioester bond (alternative pathway). All three pathways converge at the level of the C3 convertase, and induce a similar set of bioactive split products C3a, C3b, C5a, and the terminal product C5b-9, also called the membrane attack complex (MAC) (9). Regulation of the complement cascade is crucial because otherwise uncontrolled activation leads to organ damage and chronic inflammatory (autoimmune) renal diseases, including atypical haemolytic uraemic syndrome (aHUS), C3 glomerulopathy (C3G), systemic lupus erythematosus (SLE) and transplant rejection (10).

In transplantation the complement cascade can be activated by various triggers. In kidney transplantation, ischemia reperfusion injury (IRI) can cause release of damage-associated molecular patterns (DAMPS) that trigger activation (11). Later after transplantation, the presence of donor specific antibodies can result in classical pathway activation and thereby increase the risk for allograft rejection (12, 13). Also transplantation of tissues like kidney organoids are at risk of inducing deleterious complement activation, illustrated by the islet transplantation model of Xiao et al., who showed that deposition of complement product C3b in the transplanted tissue was associated with rejection (14). Complement activation is able to induce direct allograft injury *via* the formation of the MAC, and indirectly *via* the production of active split products that stimulate both the innate and adaptive immune response (15). Especially anaphylatoxins C3a and C5a contribute significantly to allograft rejection by promoting leukocyte infiltration (mainly macrophages) (16, 17) and stimulating effector T cell survival (18), proliferation and activation (17, 18) *via* signaling through complement receptors on leukocytes and tissue resident cells (16). The broad influence of complement activation on the whole immune response underscores the potential as a target for immune modulation. Although systemic complement inhibition may expose patients to higher risk of infection, genetic modification of the transplanted tissue by expression of complement inhibitors would offer site-specific protection. Multiple natural regulators of the complement system have been identified, of which many interfere at the level of the C3 convertase (9). C3 convertase is a strategic target since it is the converging point of the different activation routes and the central driver of the cascade. Inhibition of C3 convertase shuts down the complete cascade, including the production of anaphylatoxins C3a and C5a, and formation of the MAC. The effectiveness of C3 inhibition is illustrated by a recent study where compstatin was used in a kidney transplantation model in non-human primates. Improved graft survival and kidney function were achieved together with decreased macrophage infiltration, a lower amount of circulating cytokines and decreased T and B cell proliferation and activation (19).

In the current study we evaluated the potential of complement regulator CD55, also known as decay accelerating factor (DAF), which acts as a complement regulatory protein by accelerating the decay of C3 convertase and preventing its reassembly (20). Expression of CD55 in renal allografts was shown to have a protective effect on graft survival and function (21). We genetically modified iPSCs to overexpress CD55 and evaluated the complement inhibitory potential in iPSCs and iPSC-derived kidney organoids. We show that CD55 is able to inhibit complement activation and is therefore a promising candidate in the development of hypoimmunogenic iPSCs for transplantation purposes.

## Materials and methods

### iPSC maintenance and kidney organoid differentiation

Human iPSCs used in this study were generated by the LUMC iPSC Hotel using RNA Simplicon reprogramming kit (Millipore) (LUMC0072iCTRL01, detailed information at Human Pluripotent Stem Cell Registry, https://hpscreg.eu/). iPSCs were cultured on recombinant human vitronectin in Essential 8 (E8) medium (Thermo Fisher Scientific) and passaged every 3-4 days using 0.5 mM UltraPure EDTA (Thermo Fisher Scientific). iPSCs were differentiated to kidney organoids following a previously described protocol (1, 3). In short, iPSCs were incubated for 4 days in STEMdiff APEL2 medium (APEL2) containing 1% PFHMII (Life Technologies), 1% Antibiotic–Antimycotic (Life Technologies) and 8 µM CHIR99021 (Tocris). On day 4, the medium was changed to APEL2 with 200 ng mL^-1^ rhFGF9 (R&D Systems) and 1 µg mL^-1^ heparin (Sigma-Aldrich). On day 7, cells were pulsed with 5 µM CHIR for 1 hour, dissociated and transferred as small 3-dimensional clumps containing 5 × 10^5^ cells on Transwell 0.4 mm pore polyester membranes (Corning) in APEL2 containing FGF-9 and heparin. Medium was refreshed on day 7 + 3. On day 7 + 5 growth factors were removed and the APEL2 medium was refreshed every 2 days for the remaining culture time until day 7 + 14.

### Genetic modification by CRISPR-Cas9

Genetic modification of iPSCs was performed by transfection of DNA plasmids using Lipofectamine Stem Transfection Reagent (Invitrogen). iPSCs were passaged the day before transfection. For transfection 7.5 µL of Lipofectamine Stem (Thermo Fisher Scientific) was mixed with 3 µg DNA plasmids in 125 µl optiMEM and after 15 min incubation at room temperature added to iPSCs. After transfection, iPSCs were kept in culture with daily change of media for 4 days. On day 4 iPSCs were dissociated into a single-cell suspension using TrypLE select (Thermo Fisher Scientific) and 20 × 10^3^ cells per cm^2^ were transferred to new culture plates in E8 containing RevitaCell Supplement (Thermo Fisher Scientific) and 0.2 µg mL^-1^ puromycin (InvivoGen). Selection by puromycin was continued for 6 – 7 days after which plain E8 medium was added. Remaining colonies were kept in culture until they had reached a size of 2-3 mm in diameter which took roughly 10 – 13 days after the single cell passage. Single colonies were scraped off, submerged in 0.5 mM EDTA for 5 min at 37°C, washed and transferred to a new plate containing E8 medium. iPSC clones were propagated, cryopreserved and cellular material was collected to validate the genetic modification.

### Plasmid production

In each transfection for genetic modification by CRISPR-Cas9, 3 plasmids were used for homology directed repair in the Adeno-Associated Virus Integration Site 1 (AAVS1) region. 1. plasmid containing the sequence for Cas9 driven by a CAG promoter (Cas9 plasmid), 2. plasmid containing the gRNA (gRNA plasmid), and 3. plasmid containing the donor DNA for homologous recombination (donor DNA plasmid). The generation of the Cas9 plasmid (AV62_pCAG.Cas9.rBGpA) (22) and the gRNA plasmid targeting the safe harbor human locus AAVS1 (23), have been detailed elsewhere. The 2 donor DNA plasmids were assembled by inserting either an ORF for GFP or CD55 in an Age1+Not1 enzymatically digested AAVS1-CAG-puroR-T2A acceptor plasmid. The final donor DNA plasmids (pdonor.AAVS1-CAG-puroR-T2A-GFP and pdonor.AAVS1-CAG-puroR-T2A-CD55) contain AAVS1 homology arms flanking a CAG promoter, followed by the ORF of puromycin resistance gene and GFP or CD55 separated by a T2A sequence. Transformation of plasmids was performed in chemo-competent E.Coli bacteria and plasmids were isolated using maxiprep (Genomed). Correct plasmid sequences were validated by sanger sequencing by the Leiden Genome Technology Center (LGTC).

### DNA isolation and genomic PCR

DNA isolation was done on cell pellets of roughly 500 x 10^3^ iPSCs using the DNeasy kit (Qiagen) and DNA concentrations measured with nanodrop. PCR reactions were performed using GoTaq^®^ G2 Flexi DNA Polymerase (Promega) under the following conditions: 100-200 ng DNA, 1× Green GoTaq Flexi buffer, 1.25 units GoTaq G2 Flexi polymerase, 0.3 μM each primer, 0.3 mM each dNTP and 1.5 mM MgCl_2_ in a 30 μl volume for 30-40 cycles in a C1000 Touch Thermal Cycler (Bio-Rad, Hercules, USA). Primers used for screening are described in **Supplemental Table 1**. PCR products were loaded on a 1% agarose gel for size separation and DNA bands were visualized by ethidium bromide detection in a Molecular Imager Gel Doc XR+ (Bio-Rad). Size was estimated by comparing with GeneRuler DNA Ladder mix (Thermo Scientific).

### mRNA isolation and qPCR

Total RNA was isolated using a nucleospin RNA/protein kit (Bioké) and RNA concentrations were measured with nanodrop. SuperScript III Reverse Transcriptase (Invitrogen) was used for the production of cDNA that was used as a template in real-time polymerase chain reaction (RT-qPCR). RT-qPCR was performed using SYBR Green Supermix (Bio-Rad) in a CFX Connect Real-Time System (Bio-Rad). Primers used in RT-qPCR are described in **Supplemental Table 1**. The expression of each gene was normalized to the level of glyceraldehyde-3-phosphate dehydrogenase (GAPDH) expression and relative mRNA levels were determined based on the comparative Ct method (2^-ΔΔCt^) to the respective controls.

### Immunohistochemistry

Organoids were fixed in 2% PFA for 20 min at 4°C, washed with PBS and incubated with the following primary antibodies: sheep anti-human NPHS1 (1:100, AF4269, R&D Systems), mouse anti-E-Cadherin (1:250, 610181, BD), biotinylated Lotus Tetragonolobus Lectin (LTL) (1:300, B-1325, Vector Laboratories) and mouse anti-human CD55 (1:500, ab1422, Abcam). Secondary antibodies were donkey anti-sheep Alexa-647 (A21448, Invitrogen), donkey anti-mouse Alexa-568 (A10037, Invitrogen), donkey anti-mouse Alexa-488 (A21202, Invitrogen), and Streptavidin conjugated with Alexa-532 (S11224, Invitrogen), all at 1:500 dilution. Nuclei were stained with Hoechst33342 (1:10000, H3576, Invitrogen) and organoids were mounted using ProLong Gold Antifade Mountant (Invitrogen) in glass bottom dishes (MatTek). Images were acquired using a White Light Laser Confocal Microscope TCS SP8 and LAS-X Image software.

### *In vitro* complement activation assay

iPSCs or kidney organoids were stimulated with 500 IU mL^-1^ recombinant human IFN-γ (Invitrogen) 48 hours prior to the complement activation assay to increase HLA surface expression. iPSCs were dissociated into single cells using TrypLE select for 5 min at 37°C. Kidney organoids were dissociated to single cells by incubation in collagenase I buffer consisting of 600 U mL^-1^ collagenase Type I (Worthington) and 0.75 U mL^-1^ DNAse (Sigma Aldrich) in HBSS with calcium and magnesium (Thermo Fisher Scientific) for 40 minutes at 37°C with repeated pipetting, followed by TrypLE buffer consisting of 5 U mL^-1^ DNAse I (Sigma Aldrich) and 4 µg mL^-1^ heparin (Sigma Aldrich) in 80% TrypLE select 10x (Thermo Fisher Scientific) in DPBS (Thermo Fisher Scientific) for 5 min at 37°C. Dissociation was stopped by adding HBSS with 10% FBS. Cell clusters were removed by passing the cells over a 30 μm filter and the single cells were resuspended in PBS + 0,1% BSA. In the *in vitro* complement activation assay, iPSCs and kidney organoid cells were first incubated with a human anti- HLA-A2 (SN607D8) or human anti-HLA-A1 (GV2D5) antibody, generated as described previously (24) at 3 µg mL^-1^ for 30 min on ice. Normal human serum (NHS) was used as complement source and heat-inactivated normal human serum (dNHS), 45 min incubated at 56°C, was used as complement inactivated control. Cells were washed and incubated in 10% NHS or dNHS diluted in RPMI for 1 hour at 37°C, after which cells were processed for analysis by flow cytometry.

### Flow cytometry

For flow cytometry the following antibodies were used: PE-conjugated mouse anti- HLA-ABC (1:100, 555553, BD Biosciences), human anti- HLA-A2 antibody (3 µg mL^-1^, SN607D8), mouse anti-human CD55 (1:500, ab1422, Abcam), mouse anti-C3 (1:1000, RFK22, in-house generated), mouse anti-C5b-9 (1:100, AE11, Hycult Biotech), PE-conjugated mouse anti-human CD46 (1:100, 12-0469-42, Invitrogen), and APC-conjugated mouse anti-human CD59 (1:100, 17-0596-42, Invitrogen) were used as primary antibodies. Secondary antibodies used for detection were PE-conjugated goat anti-human IgG (1:1000, 109-116-098, Jackson) and PE-conjugated goat anti-mouse (1:100, R0480, DAKO). All antibody incubations were done for 30 min on ice with subsequent washing in PBS + 0,1% BSA. Flow cytometry was performed on a LSR-II (BD) and acquired results analyzed using FlowJo (BD).

### Statistical analysis

Results were visualized and statistically analyzed using Graphpad Prism. Each sample was compared to the respective control by using T-test or one-way ANOVA with Dunnett correction for multiple comparisons as indicated. Values are shown as mean + SD and p-value < 0.05 was considered statistically significant.

## Results

### iPSCs are sensitive to antibody-induced complement activation

**Figure 1:**
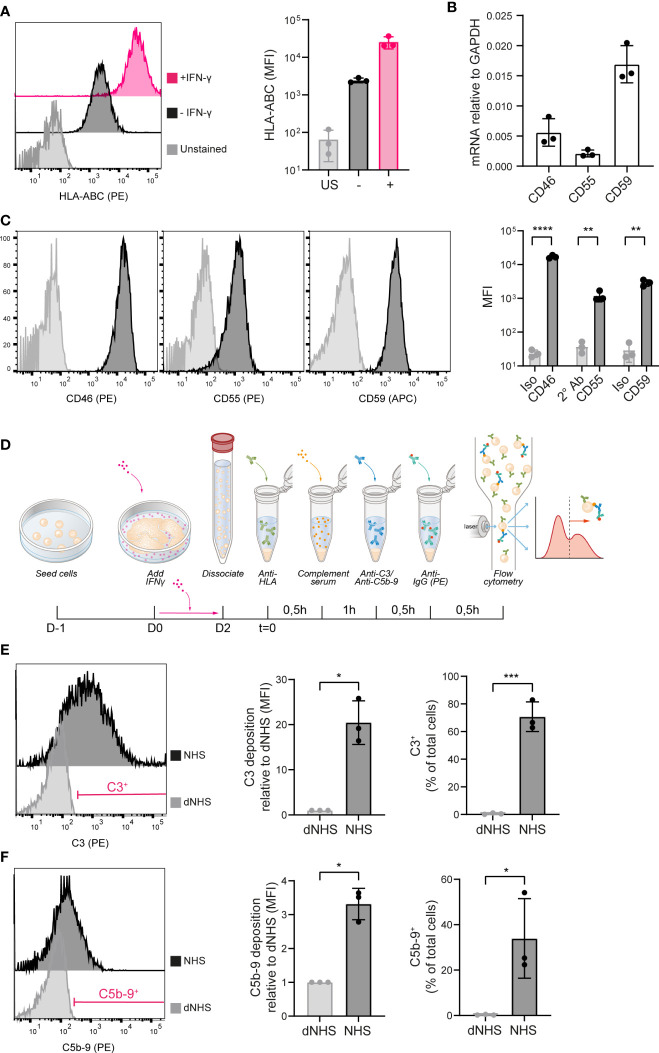
iPSCs are vulnerable to complement activation. **(A)** HLA-ABC surface protein expression measured by flow cytometry in unstained (US), unstimulated (–) and IFN-γ stimulated (+) iPSCs. A representative histogram is shown of three independent experiments (n=3). **(B)** mRNA expression of CD46, CD55 and CD59 in iPSCs measured by RT-qPCR (n=3). **(C)** Complement inhibitor CD46, CD55 and CD59 protein surface expression in iPSCs measured by flow cytometry. Representative histograms are shown of three independent experiments (n=3). **(D)** Schematic of the experimental procedure to measure complement deposition on iPSCs *in vitro*. **(E)** C3 deposition on iPSCs incubated with heat-inactivated (dNHS) or active normal human serum (NHS) measured by flow cytometry. Results are presented as relative mean fluorescence intensity (MFI) and C3^+^ proportion. A representative histogram of three independent experiments (n=3) is shown and indicates the gate for the C3^+^ proportion. **(F)** C5b-9 deposition on iPSCs incubated with heat-inactivated (dNHS) or active normal human serum (NHS) measured by flow cytometry. Results are presented as relative mean fluorescence intensity (MFI) and C5b-9^+^ proportion. A representative histogram of three independent experiments (n=3) is shown and indicates the gate for the C5b-9^+^ proportion. Error bars show standard deviation and significance (*p < 0.05; **p < 0.01; ***p < 0.001; ****p < 0.0001) was evaluated using T-test.

To evaluate the immunogenicity of iPSCs and the possible involvement of humoral immunity, we developed a system to study allo-antibody induced complement activation. iPSCs were shown to express HLA class I, which could be further increased by 48 hours incubation with IFN-γ (**Figure 1A**). iPSCs were further evaluated for expression of transmembrane complement regulators. iPSCs showed mRNA expression of the C3-convertase inhibitors CD46/MCP, CD55/DAF and the C5b-9 inhibitor CD59 (**Figure 1B**), as well as surface protein expression (**Figure 1C**).

To investigate complement activation at the surface, the HLA-A2-positive iPSCs were stimulated by IFN-γ and incubated with a human anti-HLA-A2 antibody. This was followed by exposure to complement active NHS, thereby mimicking classical pathway activation. Following 1 hour incubation at 37°C, iPSCs were analyzed for the deposition of complement activation fragments using flow cytometry (**Figure 1D**). 1 hour incubation with NHS did not affect the phenotype of the iPSCs (**Supplemental Figure 1A**), but did result in the deposition of C3 as the central complement component (**Figure 1E**) and C5b-9 as the end product of the terminal pathway (**Figure 1F**). Both the proportion positive cells as well as the MFI showed a significant increase compared to the negative control, using dNHS. Complement deposition was most prominent for C3 (mean 20 fold increase in MFI), implying an insufficient regulation of the C3-convertase on the iPSCs. In addition, we showed that incubation of iPSCs with an anti-HLA-A1 antibody, an alloantigen not expressed on these iPSCs, did not result in complement activation, indicating an alloantigen-specific response (**Supplemental Figure 1B**).

### Generation of iPSCs with stable overexpression of CD55

**Figure 2:**
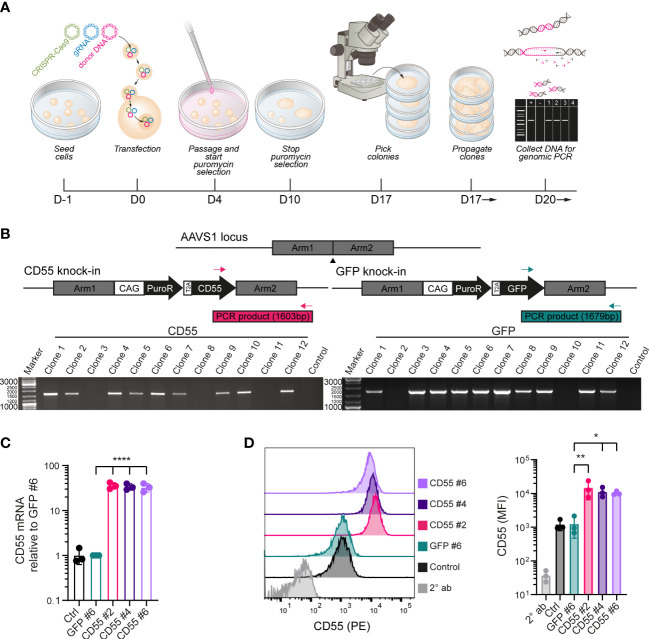
Genetic modification of iPSCs to induce over-expression of CD55. **(A)** Schematic of the genetic modification strategy by transfection of plasmids in iPSCs using lipofectamine and puromycin selection. **(B)** PCR strategy with inserted sequences for CD55 or GFP and a puromycin resistance (puroR) gene in the AAVS1 locus and analysis of puromycin selected iPSC clones with 2 distinct primer sets. DNA of the parental unmodified cell line was used as a negative control and clones were screened for the amplification of a PCR product with the indicated size (1603bp for CD55 insert or 1679bp for GFP insert). **(C)** mRNA expression of CD55 in control iPSCs and modified iPSC clones (GFP #6, CD55 #2, 4 and 6) measured by RT-qPCR (n=3). **(D)** CD55 protein expression in control iPSCs and modified iPSC clones measured by flow cytometry (n=3) showing a representative histogram. Staining with only PE-conjugated secondary antibody (2° ab) is shown in light grey. Error bars show standard deviation and significance (*p < 0.05; **p < 0.01; ****p < 0.0001) was evaluated using one-way ANOVA comparing each sample to iPSC-GFP with Dunnett correction for multiple comparisons.

iPSCs were genetically modified to (over-)express either complement inhibitor CD55 or GFP. Following transfection and selection, CRISPR/Cas9 modified iPSC clones were selected and further characterized (**Figure 2A**). To ensure stable transgene expression, we selected a synthetic CAG promoter to drive CD55 or GFP expression and the adeno-associated virus integration site 1 (AAVS1) locus as target location. The insert sequence further contained a puromycin resistance (puroR) gene to allow for selection (**Figure 2B**).

In total 12 clones were selected for both CD55 and GFP modification. AAVS1 specific locus integration was assessed by PCR using primer sets spanning the homology arm and confirmed that 9 out of 12 CD55 clones (iPSC-CD55) and 10 out of 12 GFP clones (iPSC-GFP) were modified correctly (**Figure 2B**). iPSC-CD55 clone 2, 4 and 6, and iPSC-GFP clone 6 were selected for follow-up experiments, in which iPSC-GFP served as control to test the effectivity of CD55 overexpression.

CD55 gene and protein expression were validated by qPCR and flow cytometry analysis respectively and compared to iPSC-GFP and the parental (unmodified) iPSCs, indicated as control. CRISPR-Cas9 gene editing led to consistent 30-fold increase in CD55 mRNA expression in the iPSC-CD55 clones, compared to controls (**Figure 2C**). In line with this, also CD55 protein surface expression was on average 10 – 15 times higher in all three CD55-clones compared to iPSC-GFP (**Figure 2D**). As expected, GFP expression was exclusively detected in iPSC-GFP (**Supplemental Figure 2**).

### CD55 overexpressing iPSCs are protected against complement activation

**Figure 3:**
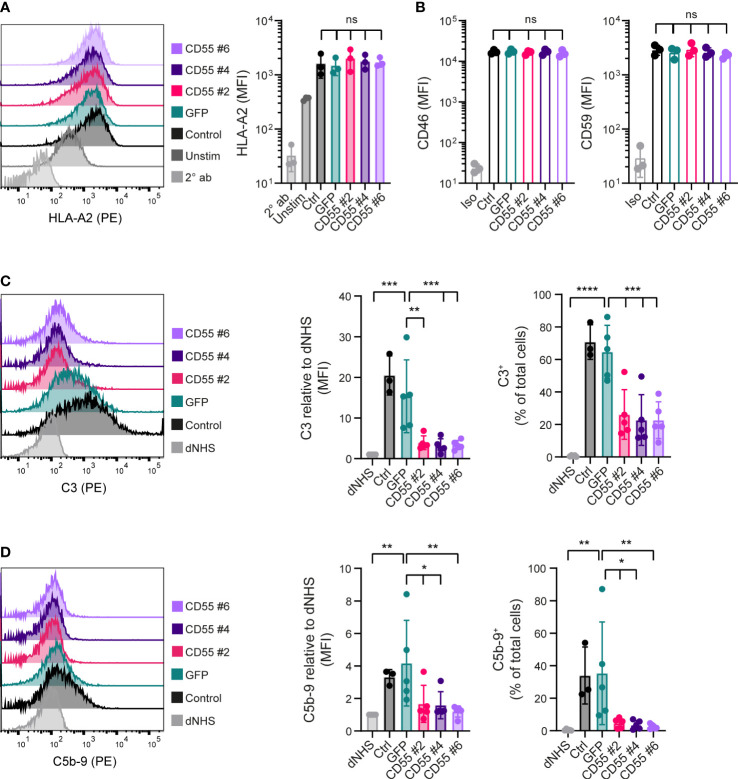
CD55 overexpression in iPSCs decreases complement deposition. **(A)** Cell surface opsonization by anti-HLA-A2 in unstimulated (Unstim) and IFN- γ stimulated (Ctrl, GFP, CD55 #2, 4 and 6) iPSCs measured by flow cytometry. A representative histogram is shown and results are presented as mean fluorescence intensity (MFI) in three independent experiments (n=3). Staining with only PE-conjugated secondary antibody (2° ab) is shown in light grey. **(B)** CD46 and CD59 protein surface expression on iPSCs measured by flow cytometry (n=3). Staining with isotype (Iso) is shown in light grey. (**C, D**) C3 and C5b-9 deposition on iPSCs incubated with normal human serum (NHS) measured by flow cytometry. Incubation with heat-inactivated NHS (dNHS) was used as negative control and is shown in light grey. Results are presented as relative mean fluorescence intensity (MFI) and C3^+^ or C5b-9^+^ proportion. A representative histogram is shown (n=3 for control, n=5 for others). Error bars show standard deviation and significance (*p < 0.05; **p < 0.01; ***p < 0.001; ****p < 0.0001) was evaluated using one-way ANOVA comparing each sample to iPSC-GFP with Dunnett correction for multiple comparisons. ns, non significant.

To evaluate whether CD55 surface overexpression affected complement activation, we used the same *in vitro* complement activation assay (**Figure 1**). iPSCs were maintained for 2 days in presence of IFN-γ to increase HLA surface expression. We confirmed that the CD55 and GFP-overexpressing clones showed a similarly increased expression of HLA-A2 (**Figure 3A**). In addition, also mRNA and surface expression of the complement regulators CD46 and CD59 were not affected by the gene modifications (**Figure 3B**; **Supplemental Figure 3**).

Complement C3 deposition at the surface of iPSC-GFP was comparable to the unmodified iPSCs. In contrast, all three CD55 overexpressing clones showed a significant inhibition of C3 deposition at the surface of these cells (**Figure 3C**), reducing it to the levels observed with dNHS. Similar results were observed for the deposition of C5b-9 (**Figure 3D**), albeit the level of C5b-9 deposition under control conditions was much lower. These data confirmed that CD55 overexpression is able to control complement activation at the surface of these iPSCs.

### CD55 overexpressing iPSCs successfully differentiate to kidney organoids

**Figure 4:**
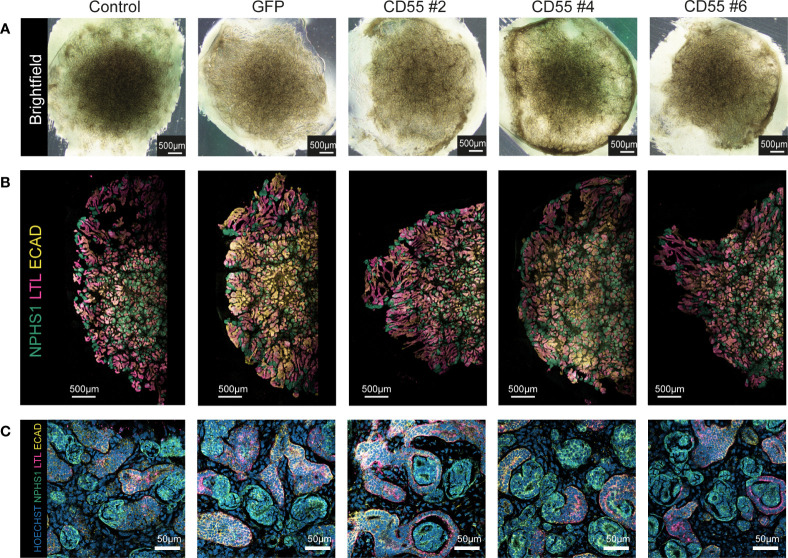
Modified iPSC-derived kidney organoids contain nephron structures upon differentiation. **(A)** Representative brightfield images of kidney organoids from unmodified iPSCs (control) and genetically modified iPSCs at day 7 + 14 of differentiation. **(B)** Representative fluorescent images of half an organoid, with detection of NPHS1 (podocytes, green), LTL (proximal tubule, pink), and ECAD (distal tubule, yellow). **(C)** Representative images of a higher magnification of the staining shown in **(B)** with additional detection of nuclei (Hoechst, blue).

Next, we differentiated the iPSC clones to kidney organoids and evaluated their ability to differentiate following genetic modification and compared their phenotype to the parental (unmodified) iPSC line (control). Macroscopic pictures showed a conserved phenotype in all the modified iPSC-CD55 and iPSC-GFP clones compared to unmodified iPSCs (**Figure 4A**). Immunohistochemical staining confirmed the presence of glomerular (NPHS1), proximal tubular (LTL) and distal tubular structures (ECAD) in all organoids (**Figures 4B, C**), demonstrating that kidney organoid differentiation was successful following genetic modification and overexpression of CD55.

### Overexpression of CD55 on differentiated kidney organoid cells protects against anti-HLA-induced complement activation

**Figure 5:**
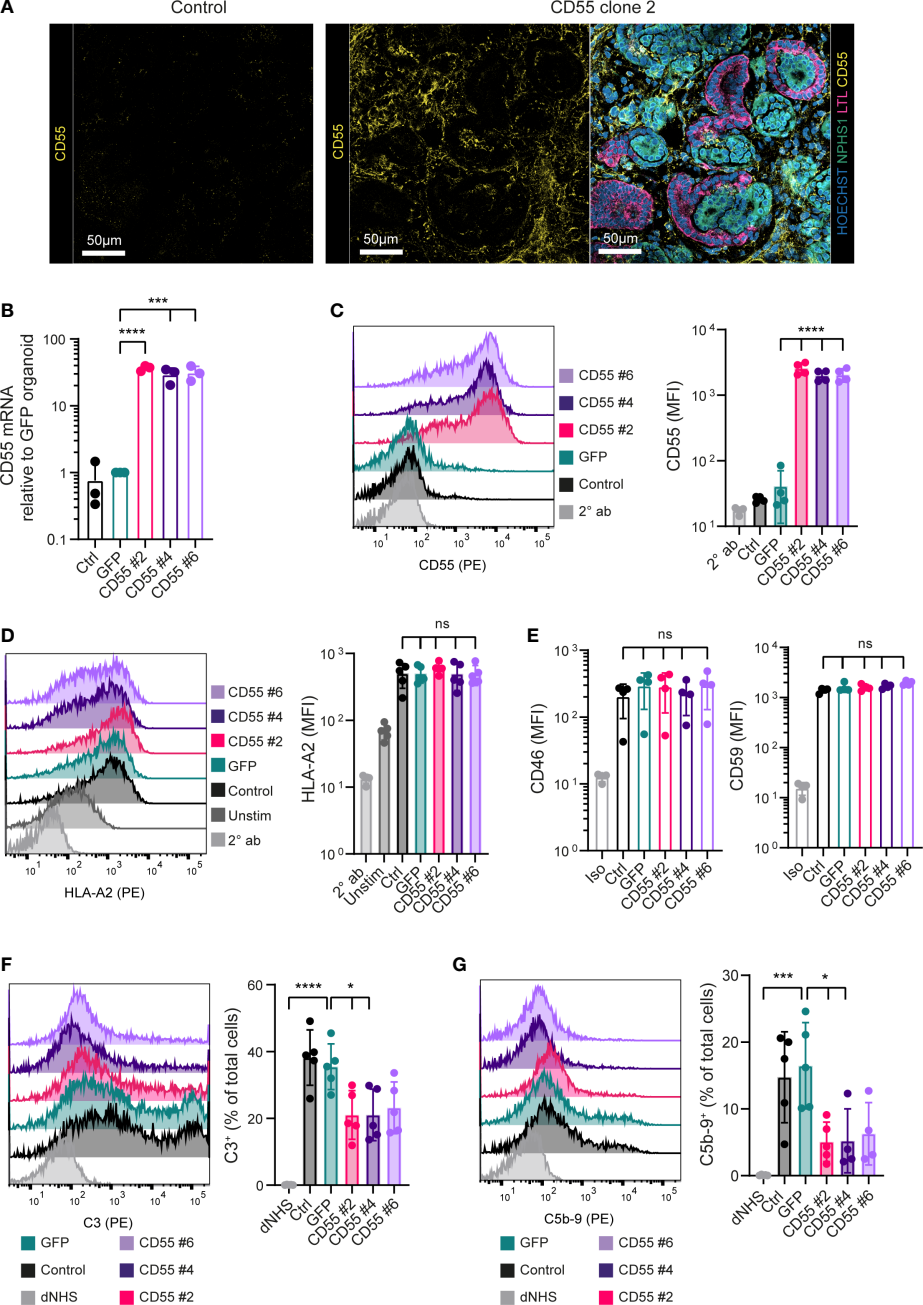
CD55 overexpression in kidney organoids decreases complement deposition. **(A)** Representative fluorescent images of organoids with detection of CD55 (yellow) in high magnification. For the CD55 #2 organoid an image with detection of Hoechst (blue), NPHS1 (green), LTL (pink) and CD55 (yellow) of the same area as the separate CD55 image is included. Other clones are included in **Supplemental Figure 4**. **(B)** CD55 mRNA expression in organoids at day 7 + 14 measured by RT-qPCR (n=3). **(C)** CD55 protein expression in dissociated kidney organoids at day 7 + 14 measured by flow cytometry showing a representative histogram of 5 independent experiments (n=5). Staining with only PE-conjugated secondary antibody (2° ab) is shown in light grey. **(D)** Cell surface opsonization by anti-HLA-A2 in unstimulated (Unstim) and IFN-γ stimulated (Ctrl, GFP, CD55 #2, 4 and 6) kidney organoid cells measured by flow cytometry. A representative histogram is shown of 5 independent experiments (n=5) and staining with only PE-conjugated secondary antibody (2° ab) is shown in light grey. **(E)** CD46 and CD59 protein surface expression on kidney organoid cells measured by flow cytometry (n=5). Staining with isotype (Iso) is shown in light grey. (**F**, **G**) C3 and C5b-9 deposition on kidney organoid cells incubated with normal human serum (NHS) measured by flow cytometry. Incubation with heat-inactivated NHS (dNHS) was used as negative control and is shown in light grey. Results are presented as C3^+^ or C5b-9^+^ proportion. A representative histogram is shown of 5 independent experiments (n=5). Error bars show standard deviation and significance (*p < 0.05; ***p < 0.001; ****p < 0.0001) was evaluated using one-way ANOVA comparing each sample to iPSC-GFP-derived kidney organoids with Dunnett correction for multiple comparisons. ns, non significant.

Control kidney organoids showed low expression of CD55, as investigated by immunofluorescence staining (**Figure 5A**). On the other hand, CD55 overexpressing clones showed prominent CD55 staining throughout the organoid (**Figure 5A**; **Supplemental Figure 4A**). In line with the immunofluorescence analysis, the CD55 mRNA expression was low in control organoids, but strongly increased in the CD55 overexpressing clones (**Figure 5B**). This was confirmed by the difference in CD55 surface expression as shown by flow cytometry (**Figure 5C**). It should be noted that the CD55 expression was more heterogenous compared to the expression on iPSCs. Similar to CD55, GFP expression was maintained after differentiation of GFP-iPSCs towards kidney organoids (**Supplemental Figures 4B, C**).

To assess functionality of CD55 overexpression, we adapted the protocol used for iPSCs to measure complement deposition on single organoid cells. Kidney organoid cells showed an increased expression of HLA-A2 by IFN-γ stimulation (**Figure 5D**) and CD55 overexpressing clones showed a similar expression of CD46 and CD59 as control and iPSC-GFP-derived kidney organoids (**Figure 5E**; **Supplemental Figure 5**). When exposed to anti-HLA-A2 antibodies, followed by NHS, organoid cells showed a significant increase in complement activation, as shown by the deposition of C3 and C5b-9, compared to the negative control using dNHS (**Figures 5F, G**). Complement deposition was significantly inhibited in the CD55-overexpressing clones. All together these data show that CD55 overexpression can be an efficient tool to diminish antibody-mediated complement activation, both in undifferentiated iPSCs as well as differentiated kidney organoids.

## Discussion

Since the generation of iPSCs (25, 26), the application of their derived tissues in regenerative medicine holds great expectations for solving the shortage of donor organs. Yet, immune surveillance of the host remains a barrier for further clinical applications. In this study we demonstrate the potential of CD55 overexpression in iPSCs and differentiated kidney organoids to improve resistance towards complement activation. Importantly, we show that knock-in of CD55 in the AAVS1, ‘safe harbour’ (27), locus did not influence the quality of the kidney organoids and allowed a stable and strong CD55 expression throughout the differentiation.

Our data illustrate a different sensitivity of iPSCs compared to kidney organoids to complement activation, shown by a higher intensity of complement deposition on differentiated cells. This observation underscores the importance of better control on complement activation when iPSC-derived cells and tissues are considered to be used in transplantation. Interestingly we found reduced CD55 expression during kidney organoid differentiation (**Figures 1D**, **5C**), which is in line with our previous work on embryonic stem cell-derived beta cells that was accompanied with reduced CD55 expression (28). In contrast, complement regulatory proteins, CD46 and CD59, remained present following kidney organoids differentiation (**Supplemental Figure 5**). Therefore the low surface CD55 expression detected in the kidney organoids may play a more prominent role in the increased vulnerability to complement activation and overexpression of CD55 provides the opportunity to increase resistance. CD55 knock-in iPSCs can be used for differentiation of any desirable tissue or cell type. Although we found that the complement inhibition by CD55 overexpression was evident for both undifferentiated iPSCs and iPSC-derived kidney organoids, this can not directly be translated to all differentiated cell types. Further research is necessary to validate the use of CD55 overexpression in other iPSC-derived cells.

In our studies on complement reactivity to iPSC-derived kidney organoids we focused on analysis of dissociated cells. The use of flow cytometry to evaluate complement activation provides higher sensitivity and offers the possibility for quantification. For future research however, it would be valuable to extend the complement reactivity and apply the experimental setup to whole organoids. Additionally it would be of critical importance to validate the findings in an *in vivo* situation, which could also allow for the evaluation of the interaction with other immune components.

The use of CD55, which intervenes in the complement cascade at C3 convertase, is strategically chosen, since it targets the converging point of all three complement activation routes. Our results showed that CD55 overexpression inhibited deposition of both C3 and C5b-9, confirming that the complement cascade was inhibited from C3 to the end of the cascade where the C5b-9 complex, or MAC, is formed. Moreover, overexpression of CD55 may have additional beneficial effects that we did not investigate here (20), since other immune regulation effects have been found including the regulation of T cell proliferation and activation *via* CD97 binding (29), and reduction of NK cell reactivity (30).

In our study, CD55 overexpression did not fully prevent complement deposition, raising the question if additional inhibitory molecules are required. The necessary degree of complement protection is greatly dependent on the application of the transplantable tissue. Intraportal transplantation, commonly used for islet transplantation in patients with type I diabetes, involves a great impact of complement activation as part of the immediate blood mediated immune reaction (IBMIR), and might need a higher grade of protection (31, 32). Furthermore, combination of multiple complement regulatory proteins has shown additive effect in other conditions. In mice it was shown that CD55 is protective against IRI and co-expression with CD59 provided additional protection (33). The need for combining complement regulators is further illustrated by the recent developments in the field of xenotransplantation. For instance higher CD55 and CD59 expression in pig fibroblasts increased their resistance towards human serum mediated cytolysis (34). Similarly in cytotoxicity assays using porcine peripheral blood mononuclear cells of genetically modified pigs, the combination of CD55 and CD59 offered higher protection than either of them alone (35). Also in experimental xenotransplant models, genetic modifications were applied to pigs to overcome these immunological barriers and the addition of complement regulatory proteins CD46, CD55 and/or CD59 was advantageous for transplant outcome (35–37). This has resulted in the first pig to human xenotransplants using 10-gene modified pigs, which include the overexpression of CD55 and CD46 (38, 39).

Complement activation can cause cytotoxicity directly but also functions as an important inducer of innate immune cell infiltration and bridges the innate with the adaptive immune response. In accordance with this, it was shown that C3 inhibition by the compstatin analog Cp40 in kidney transplantation in primates reduced macrophage infiltration, cytokine production and T and B cell activation (19). However, it is unlikely that complement inhibition alone will prevent alloimmune rejection since this study also showed progressive adaptive immune responses despite complement inhibition.

In conclusion, our results demonstrate that CD55 over-expressing iPSCs and their derived kidney organoids are less susceptible to complement activation *in vitro*, providing evidence for the use of CD55 genetic manipulation to improve transplant outcomes of iPSC-derived tissues.

## Data availability statement

The datasets presented in this study can be found in online repositories. The names of the repository/repositories and accession number(s) can be found in the article/**Supplementary Material**.

## Author contributions

LG performed the experiments, analyzed results and wrote the manuscript, RN and MD performed experiments, SH provided the anti-HLA-A1 and anti-HLA-A2 antibody, ME advised the project and reviewed the manuscript, AZ and CB supervised the project and wrote the manuscript, TJR arranged funding and supervised the project, CK supervised the project and wrote the manuscript. All authors contributed to the article and approved the submitted version.

## Supplementary material

Supplementary Figure 1 Single cell gating and alloantigen-specific complement activation**. (A**) The gating strategy for iPSCs in flow cytometry experiments is indicated for iPSCs incubated with normal human serum (NHS) and heat-inactivated NHS (dNHS). **(B**) C3 and C5b-9 deposition on IFN-γ stimulated and unstimulated (Unstim) iPSCs incubated with allo-antibodies followed by normal human serum (NHS) measured by flow cytometry (n=2). Complement deposition is compared between cells incubated with binding HLA-A2 allo-antibody (Allo ab +) and incubation with HLA-A1 (Allo ab -) that is unable to bind to the cells. Incubation with heat-inactivated NHS (dNHS) was used as negative control and error bars show standard deviation.

Supplementary Figure 2 GFP expression in modified iPSCs. **(A)** mRNA expression of GFP in control iPSCs and modified iPSC clones measured by RT-qPCR (n=3). **(B)** GFP protein expression in iPSCs measured by flow cytometry (n=3) showing a representative histogram. Error bars show standard deviation and significance (****p<0.0001) was evaluated using one-way ANOVA comparing each sample to Control iPSCs with Dunnett correction for multiple comparisons.

Supplementary Figure 3 mRNA and protein expression of complement regulatory proteins CD46 and CD59 in iPSCs. **(A)** CD46 mRNA expression in iPSCs measured by RT-qPCR (n=3). **(B)** CD46 protein surface expression measured by flow cytometry showing a representative histogram of 3 independent experiments (n=3) of which combined results are presented in **Figure 3B**. **(C)** CD59 mRNA expression in iPSCs measured by RT-qPCR (n=3). **(D)** CD59 protein surface expression measured by flow cytometry showing a representative histogram of 3 independent experiments (n=3) of which combined results are presented in **Figure 3B**. Staining with isotype (Iso) is shown in light grey. Error bars show standard deviation and significance was evaluated using one-way ANOVA comparing each sample to iPSC-GFP with Dunnett correction for multiple comparisons.

Supplementary Figure 4 CD55 and GFP expression in kidney organoids derived from modified iPSCs. **(A)** Representative fluorescent images in high magnification of organoids with detection of CD55 (yellow) in the upper panel, and an image with detection of Hoechst (blue), NPHS1 (green), LTL (pink) and CD55 (yellow) of the same area in the lower panel. **(B)** mRNA expression of GFP in kidney organoids at day 7 + 14 measured by RT-qPCR (n=3). **(C)** GFP protein expression in dissociated kidney organoids at day 7 + 14 measured by flow cytometry (n=5) showing a representative histogram. Error bars show standard deviation and significance (****p<0.0001) was evaluated using one-way ANOVA comparing each sample to Control kidney organoids with Dunnett correction for multiple comparisons.

Supplementary Figure 5 mRNA and protein expression of complement regulatory proteins CD46 and CD59 in iPSC-derived kidney organoids. **(A)** CD46 mRNA expression in kidney organoids at d7+14 measured by RT-qPCR (n=3). **(B)** CD46 protein expression in kidney organoids at d7+14 measured by flow cytometry including a representative histogram of 5 independent experiments (n=5) of which combined results are presented in **Figure 5E**. **(C)** CD59 mRNA expression in kidney organoids at d7+14 measured by RT-qPCR (n=3). **(D)** CD59 protein expression in kidney organoids at d7+14 measured by flow cytometry including a representative histogram of 5 independent experiments (n=5) of which combined results are presented in **Figure 5E**. Staining with isotype (Iso) is shown in light grey. Error bars show standard deviation and significance was evaluated using one-way ANOVA comparing each sample to iPSC-GFP-derived kidney organoids with Dunnett correction for multiple comparisons.
