# Supplementary figures and images for: Inhibition of complement activation by CD55 overexpression in human induced pluripotent stem cell derived kidney organoids
Source: Front Immunol. 2023 Jan 12;13:1058763. doi: 10.3389/fimmu.2022.1058763 (PMC9880527; doi:10.3389/fimmu.2022.1058763)

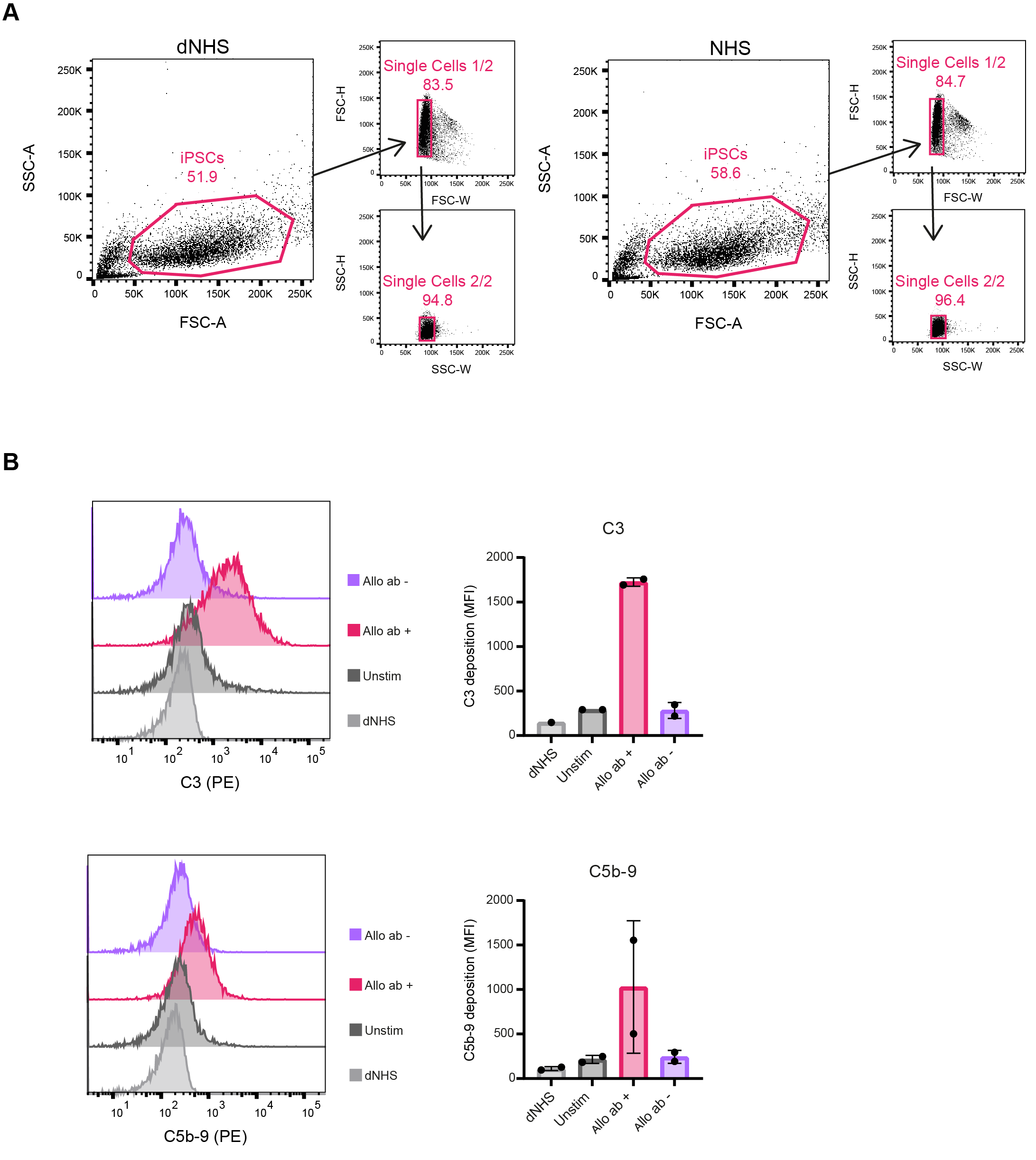

Supplement: Supplementary Figure 1 — Single cell gating and alloantigen-specific complement activation. (A) The gating strategy for iPSCs in flow cytometry experiments is indicated for iPSCs incubated with normal human serum (NHS) and heat-inactivated NHS (dNHS). (B) C3 and C5b-9 deposition on IFN-γ stimulated and unstimulated (Unstim) iPSCs incubated with allo-antibodies followed by normal human serum (NHS) measured by flow cytometry (n=2). Complement deposition is compared between cells incubated with binding HLA-A2 allo-antibody (Allo ab +) and incubation with HLA-A1 (Allo ab -) that is unable to bind to the cells. Incubation with heat-inactivated NHS (dNHS) was used as negative control and error bars show standard deviation. [file Image_1.tif]

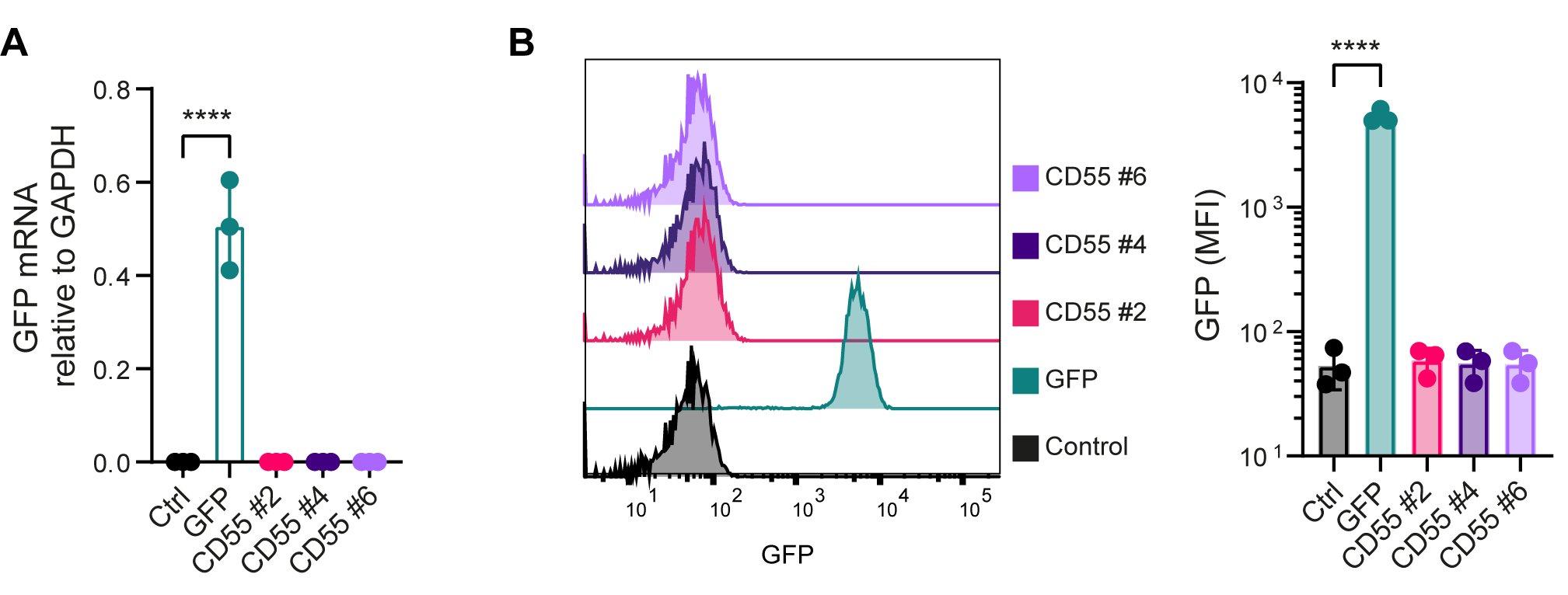

Supplement: Supplementary Figure 2 — GFP expression in modified iPSCs. (A) mRNA expression of GFP in control iPSCs and modified iPSC clones measured by RT-qPCR (n=3). (B) GFP protein expression in iPSCs measured by flow cytometry (n=3) showing a representative histogram. Error bars show standard deviation and significance (****p<0.0001) was evaluated using one-way ANOVA comparing each sample to Control iPSCs with Dunnett correction for multiple comparisons. [file Image_2.tif]

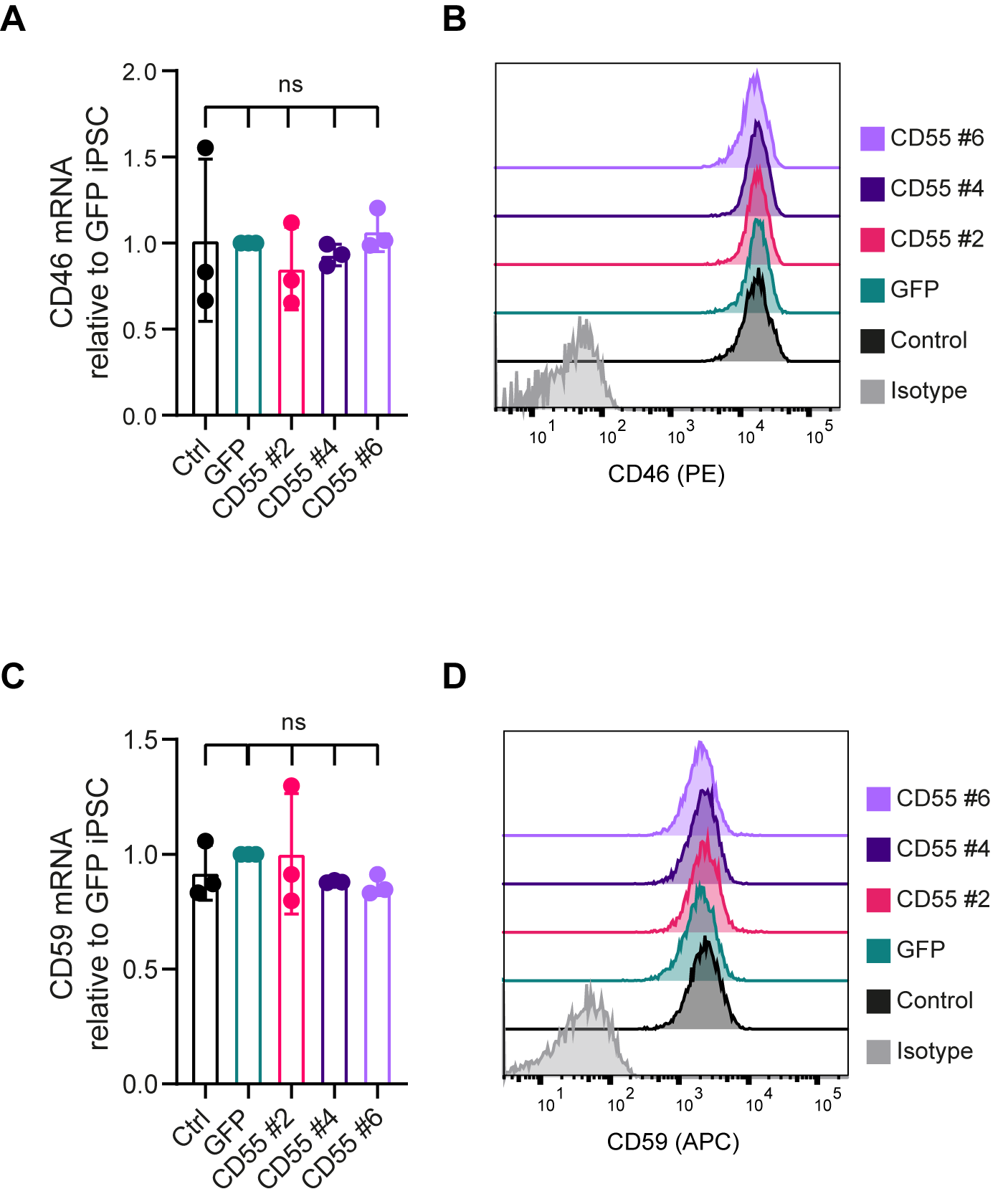

Supplement: Supplementary Figure 3 — mRNA and protein expression of complement regulatory proteins CD46 and CD59 in iPSCs. (A) CD46 mRNA expression in iPSCs measured by RT-qPCR (n=3). (B) CD46 protein surface expression measured by flow cytometry showing a representative histogram of 3 independent experiments (n=3) of which combined results are presented in Figure 3B . (C) CD59 mRNA expression in iPSCs measured by RT-qPCR (n=3). (D) CD59 protein surface expression measured by flow cytometry showing a representative histogram of 3 independent experiments (n=3) of which combined results are presented in Figure 3B . Staining with isotype (Iso) is shown in light grey. Error bars show standard deviation and significance was evaluated using one-way ANOVA comparing each sample to iPSC-GFP with Dunnett correction for multiple comparisons. [file Image_3.tif]

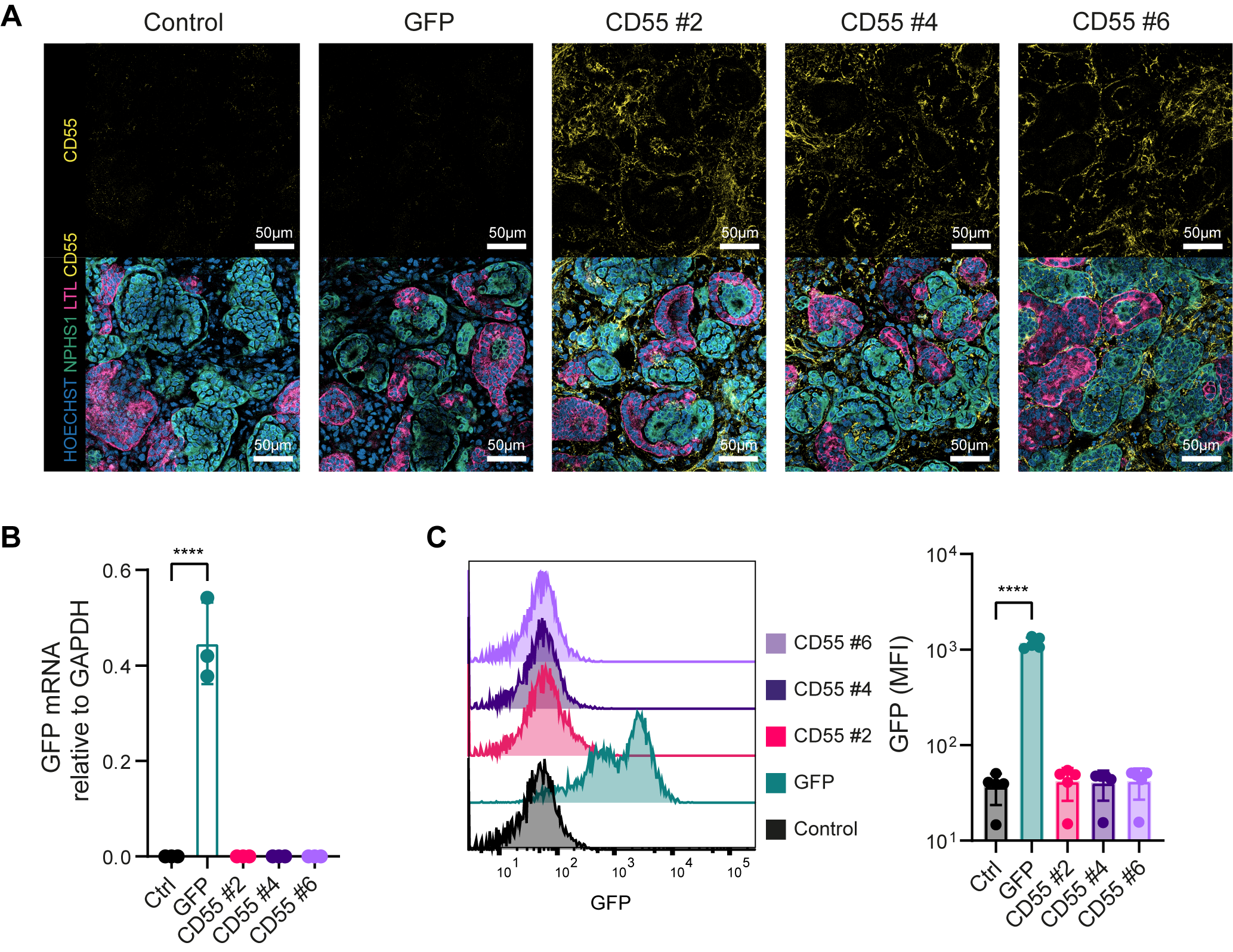

Supplement: Supplementary Figure 4 — CD55 and GFP expression in kidney organoids derived from modified iPSCs. (A) Representative fluorescent images in high magnification of organoids with detection of CD55 (yellow) in the upper panel, and an image with detection of Hoechst (blue), NPHS1 (green), LTL (pink) and CD55 (yellow) of the same area in the lower panel. (B) mRNA expression of GFP in kidney organoids at day 7 + 14 measured by RT-qPCR (n=3). (C) GFP protein expression in dissociated kidney organoids at day 7 + 14 measured by flow cytometry (n=5) showing a representative histogram. Error bars show standard deviation and significance (****p<0.0001) was evaluated using one-way ANOVA comparing each sample to Control kidney organoids with Dunnett correction for multiple comparisons. [file Image_4.tif]

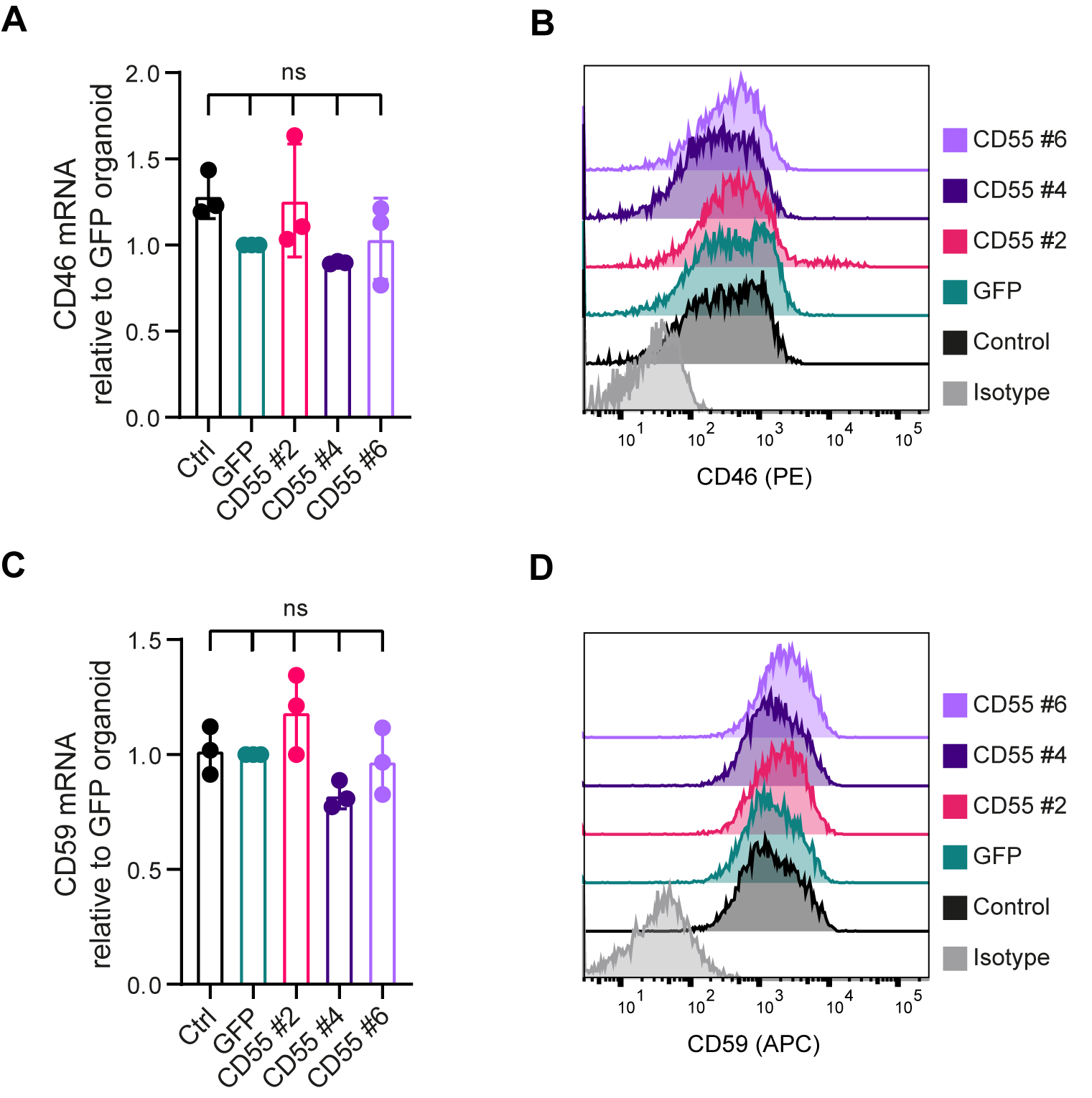

Supplement: Supplementary Figure 5 — mRNA and protein expression of complement regulatory proteins CD46 and CD59 in iPSC-derived kidney organoids. (A) CD46 mRNA expression in kidney organoids at d7+14 measured by RT-qPCR (n=3). (B) CD46 protein expression in kidney organoids at d7+14 measured by flow cytometry including a representative histogram of 5 independent experiments (n=5) of which combined results are presented in Figure 5E . (C) CD59 mRNA expression in kidney organoids at d7+14 measured by RT-qPCR (n=3). (D) CD59 protein expression in kidney organoids at d7+14 measured by flow cytometry including a representative histogram of 5 independent experiments (n=5) of which combined results are presented in Figure 5E . Staining with isotype (Iso) is shown in light grey. Error bars show standard deviation and significance was evaluated using one-way ANOVA comparing each sample to iPSC-GFP-derived kidney organoids with Dunnett correction for multiple comparisons. [file Image_5.tif]
