# Supplementary material for: Inhibition of complement activation by CD55 overexpression in human induced pluripotent stem cell derived kidney organoids
Source: Front Immunol. 2023 Jan 12;13:1058763. doi: 10.3389/fimmu.2022.1058763 (PMC9880527; doi:10.3389/fimmu.2022.1058763)
Supplement: Supplementary file 6 [file Table_1.docx]

Inhibition of complement activation by CD55 overexpression in human induced pluripotent stem cell derived kidney organoids

Supplemental material

Supplemental Table 1: Nucleotide sequences of primers used for genomic PCR and RT-qPCR.

| **Primer** | **Sequence** |
| --- | --- |
| **Genomic PCR** |  |
| CD55 ins FW | CGTTGACAGGTTTGCTTGGG |
| GFP ins FW | AACCACTACCTGAGCACCCA |
| AAVS1 dArm2 RV | TCCAGCCCCTCCTACTCTAG |
| **RT-qPCR** |  |
| GFP FW  GFP RV | ACCCCGACCACATGAAGCAGC  CGTTGGGGTCTTTGCTCAGGG |
| CD55 FW  CD55 RV | CCAAATGCTCAAGCAACACG  AAACACGTGTGCCCAGATAG |
| CD59 FW  CD59 RV | CTGCTGCAAGAAGGACCTGT  GCTGCCAGAAATGGAGTCAC |
| CD46 FW  CD46 RV | TTGTGATCCTGCACCTGGAC  TTACACTCTGGAGCAGCACG |
| GAPDH FW  GAPDH RV | ACAGTCAGCCGCATCTTCTT  AATGAAGGGGTCATTGATGG |
